# Synthesis and performance evaluation of zinc oxide tubes/alginate microfibre composites for photodegradation of methylene blue: a novel reporting approach

**DOI:** 10.1039/d4ra01229a

**Published:** 2024-06-24

**Authors:** Anas Bsoul, Ibrahim Alkhaldi, Borhan Albiss, Yusuf Selim Ocak, Mohamed Sultan Mohamed Ali

**Affiliations:** a Department of Computer Engineering, Jordan University of Science and Technology P.O. Box 3030 Irbid 22110 Jordan aabsoul1@just.edu.jo; b Institute of Nanotechnology, Jordan University of Science and Technology P.O. Box 3030 Irbid 22110 Jordan; c Department of Physics and Engineering Physics, Morgan State University Baltimore Maryland 21234 USA; d Department of Electrical Engineering, College of Engineering, Qatar University Doha Qatar

## Abstract

This research investigates the efficacy of zinc oxide (ZnO) tubes in decontaminating polluted water using a substrate-free hydrothermal synthesis process for ZnO tubes. The synthesized tubes are impregnated into calcium alginate microfibres, strategically chosen for their high surface area to enhance photocatalytic degradation performance and for practical handling during decontamination and subsequent collection, thereby preventing secondary contamination. Structural and morphological analyses, conducted using Scanning Electron Microscopy (SEM) and X-ray Diffraction (XRD), thoroughly characterize the properties of the ZnO tubes and the composite material. The efficacy of this composite is demonstrated through the photocatalytic degradation of methylene blue (MB), as a representative organic pollutant, resulting in an 88% degradation of MB after 5 hours of irradiation by a sun simulator. Cyclic tests exhibit consistent degradation levels in the first four cycles (81–89%), followed by a subsequent decrease to 72% in the fifth cycle, coinciding with the breakdown of the microfibres into shorter fragments. Innovatively, this study introduces an approach to reporting photocatalytic degradation results, utilizing normalized pollutant concentration plotted against irradiated energy instead of time, as energy encompasses irradiated power, time, and surface area. This reveals that the 88% degradation of MB is achieved by irradiating the sample with an approximately 18 kJ. Additionally, a new metric, Specific Energy Efficiency (SEE), is introduced. It expresses the ratio of degraded pollutant mass to the mass of photocatalytic active material per unit of irradiated energy, with the maximum and cumulative SEE in this study being 1.044 μg g^−1^ J^−1^ and 326 ng g^−1^ J^−1^, respectively. This research not only contributes to the understanding of ZnO tubes' efficiency in polluted water decontamination but also introduces valuable insights for standardized reporting in photocatalytic degradation studies.

## Abstract 2

The synthesis of ZnO nanotubes-alginate microfibres for MB photocatalytic degradation with innovative results presentation.

## Graphical abstract

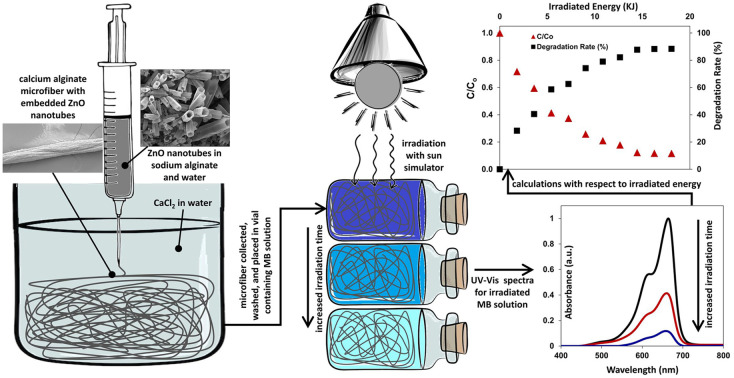

## Introduction

Water is a valuable resource, yet only a small percentage of Earth's surface water is in a suitable ecological and chemical condition. For example, less than 40% of Europe's surface water is considered safe for use.^1^ One of the main reasons for this is the increasing number of water-polluting industries that discharge contaminated waste water.^2^ These industries include, but are not limited to, the pharmaceutical, cosmetic, leather, paper, and textile industries.^2,3^ Dyes are among the top pollutants found in water, with over 700 000 tons produced annually for various industries.^4^ It is estimated that up to 25% of these dyes are lost in the waste water produced by these industries.^4,5^ As a result, dyes are believed to contribute to 17–20% of water pollution.^6^

In addition to the high toxicity of these dyes to both humans and other organisms, the limited light penetration in dyed water negatively impacts biological processes and disturbs aquatic ecosystems.^2,7,8^ Furthermore, many of these dyes have stable structures. For this reason, the traditional treatment methods are not effective at degrading them, leading to their absorption by plants and posing a risk to the food chain and human health.^5–7,9^ As a result, it is crucial to develop advanced wastewater decontamination technologies to mitigate the negative effects of these water-polluting industries.

The photocatalytic degradation of organic water pollutants is gaining significant attention as it is an environmentally friendly, low-cost, effective method for eliminating dyes, and it has the potential not to cause secondary pollution.^10,11^ Nanostructures of metal oxides are an excellent choice for photocatalytic degradation because of their excellent absorption in the UV-Vis spectrum, wide bandgap, high surface-to-volume ratio, non-toxicity, and affordability.^10,12–17^ Due to their unique properties, TiO_2_ and ZnO are considered the most important materials in this field.^18^

Despite the extensive studies on using TiO_2_ in the photocatalytic degradation of water pollutants, its effectiveness is limited by several shortcomings. TiO_2_ requires UV irradiation for photocatalytic degradation to occur due to its high bandgap.^19^ As a result, this limits its ability to utilize solar energy as UV radiation makes up less than 5% of the sunlight spectrum.^19,20^ Additionally, the fast photogenerated electron–hole pairs recombination also decreases its photocatalysis efficiency.^17,21^ Furthermore, the European Food Safety Authority (EFSA) has declared recently that TiO_2_ is not categorized among safe food additives anymore.^22^ Therefore, its usage in water decontamination poses a risk to the food chain and human health.

ZnO is gaining significant attention as an alternative to TiO_2_ in photocatalysis due to its ability to have photocatalytic activity not only under UV light but also under visible light spectrums, thanks to its inherent surface defects.^17,20^ Additionally, ZnO is more cost-effective than TiO_2_.^23,24^ However, ZnO nanostructures have been shown to lose some of their photocatalytic activity when exposed to UV light in water due to photo-corrosion.^25^ It has been reported that immobilizing ZnO nanostructures in a matrix material, such as calcium alginate or polyaniline, limits photo-corrosion and slows charge recombination,^10,25^ offering a potential solution for this limitation.

MB is a widely used dye in various industries, including printing, paper production, and textiles.^26–28^ However, improper disposal and limited water treatment technology used for industrial discharge leads to the release of MB into the environment, contributing to water pollution. While this research focuses on photodegradation of MB using ZnO, it should be considered as an example only, as ZnO has shown the capability of photodegrading many other pollutants such as malachite green, methyl violet, resazurin, RhB, acid fuchsin, and phenol.^29–33^ The research aims not only to reduce pollution caused by MB, but also to provide a broader understanding of the capability of ZnO as a photocatalyst for degradation of organic pollutants.

ZnO nanostructures, such as nanorods, nanoflowers, and nanoparticles, have been widely studied for their potential use in photocatalytic degradation of MB in water.^9,34–37^ For example, Bourfaa *et al.* synthesized both ZnO nanorods and nanoflowers on glass substrates and used them to photodegrade MB.^34^ After 300 minutes of UV irradiation, the ZnO nanoflowers and nanorods achieved 81% and 66% MB degradation, respectively. Siddiqui *et al.* developed a biocomposite of alginate and ZnO nanoparticles that displayed an impressive 98% degradation efficiency of MB after 90 minutes of UV irradiation under optimized conditions.^10^ Notably, the composite was found to be more effective at degrading MB than pure ZnO nanoparticles. Other studies have also demonstrated the effectiveness of ZnO nanostructures for MB photodegradation, including Mazzeo *et al.*^35^ who used ZnO and TiO_2_ nanoparticles embedded in calcium alginate and found that ZnO showed higher MB degradation efficiency, Muslim *et al.*^36^ also successfully photodegraded MB using ZnO nanoparticles dispersed directly into MB aqueous solution, and Abu-Dalo *et al.*^37^ fabricated polymeric membranes impregnated with ZnO nanoparticles and nanorods for the degradation of MB in water. However, a common limitation among these studies is the lack of reported experiment parameters such as irradiate surface area and the amount of used ZnO, which makes it difficult to compare performance across studies. Additionally, taking multiple samples during the course of the experiment for UV-Vis spectroscopy, can also be considered a limitation, as it makes it difficult to make reliable comparisons and understand the real performance of the photocatalysis, let alone the evaporation during the course of the study due to elevated temperature contributed to irradiation.

In contrast to previous studies that have primarily focused on ZnO nanoparticles and nanorods, our research aims to investigate the potential of ZnO tubes as a photocatalyst for the degradation of water pollutants. The improved performance of ZnO nanorods over nanoparticles in photo-degradation has been well established in the literature.^38,39^ ZnO tubes will offer better performance due to their increased surface area and added resistance to photo-corrosion.^40^ To further enhance the efficiency and practicality of our approach, the ZnO tubes are embedded in microfibre-shaped calcium alginate to serve three main purposes: (1) reducing the photo-corrosion of ZnO tubes, (2) facilitating easy filtration of the composite after the pollutant degradation to prevent secondary pollution, and (3) shaping the composite into microfibres is to increase the surface area. This composite shape ensures more exposed tubes and minimizes the distance between fully embedded tubes within the alginate and the interface of the polluted water and the microfibre. Additionally, this study reports the degraded amount of MB per amount of ZnO tubes and irradiated energy, providing a performance measurement metric that is independent of the experiment setup. MB is used as a model pollutant for this study, but the results can also be extrapolated to other pollutants as ZnO is known for the ability to photodegrade many other pollutants.^29–33^ To the best of our knowledge, there are few studies that investigate the photo-degradation of pollutants using ZnO tubes, such as Meethal *et al.*,^41^ yet none have embedded them into a matrix material like calcium alginate, which mitigates photo-corrosion and facilitates easy filtration of the composite after the pollutant is degraded. This makes our proposed approach an innovative and promising solution for the treatment of water pollutants.

## Experimental

### Materials

High-grade materials were used in all experiments, including zinc nitrate hexahydrate (Zn(NO_3_)_2_·6H_2_O, ≥99%, CAS-No: 10196-18-6), hexamethylenetetramine (C_6_H_12_N_4_, ≥99%, CAS No: 100-97-0), sodium alginate (C_6_H_7_NaO_6_, viscosity ≥150, CAS No: 9005-38-3), calcium chloride (CaCl_2_, ∼94–97%, CAS No: 10043-52-4), and methylene blue (CAS No: 7220-79-3).

### Synthesis of ZnO tubes

ZnO tubes were prepared using a surface-free hydrothermal process that was adopted and modified from the method described by Katiyar *et al.*^42^ to achieve ZnO tubes instead of ZnO nanoflowers. First, 75 mM of zinc nitrate hexahydrate (Zn(NO_3_)_2_·6H_2_O) and 75 mM of hexamethylenetetramine (C_6_H_12_N_4_) solutions were separately prepared in deionized water (DI). The solutions were then stirred at 1000 RPM for approximately 1 hour at ambient temperature. Any undissolved material was removed by filtering the solutions through a syringe filter. The hexamethylenetetramine was added slowly to the zinc nitrite hexahydrate solution while continuing to stir at 1000 RPM. The resulting solution was left to mix for an additional hour. The solution was then transferred to a sealed bottle and placed in a water bath (Isotemp SWB 15, Fisher Scientific) at an ambient temperature. The water bath was set to 90 °C and timed for 4 hours. It took the water bath around 100 minutes to reach the desired temperature. The bottle was left in the water bath until it cooled down to ambient temperature. The precipitated ZnO nanorods were left in the bottle for 3 days in the same solution they were synthesized to allow they rods' inner core to etch, forming tubes. Afterward, the resulting ZnO tubes were filtered through filter paper and washed several times with alternating cycles of ethanol and DI water to remove any by-product components. The filtered ZnO tubes were left to dry, and then they were collected as a dried powder. The chemical reactions for the synthesis of ZnO tubes are described by the following chemical reactions:^43–47^

1- Hydrolysis of hexamethylenetetramine (HMTA):

1 C_6_H_12_N_4_ + 6H_2_O → 6HCHO + 4NH_3_

2 NH_3_ + H_2_O → NH_4_^+^ + OH^−^

2- Dissociation of zinc nitrate hexahydrate:

3 Zn(NO_3_)_2_·6H_2_O → Zn^2+^ + 2NO_3_^−^ + 6H_2_O

3- Formation of zinc oxide nanorods and tubes:

4 Zn^2+^ + 2OH^−^ ↔ ZnO + H_2_O

It should be noted that eqn (3) represents both the formation of the ZnO rods and their etching into tubes. Leaving the nanorods in the growth solution allows them to etch slowly, with the highest etching rate occurring in the axial direction, ultimately leading to the formation of tubes.

### Synthesis of ZnO tubes/calcium alginate (ZnOAlg) microfibres

After the preparation of ZnO tubes, 0.75 wt% of the ZnO tubes powder was gradually added to DI water while stirring to ensure a homogeneous distribution. The mixture was left to stir continuously while 2 wt% of sodium alginate was gradually added until it was fully dissolved, and the mixture became homogeneous. The mixture of ZnO tubes and sodium alginate was then extruded through a 30 G gauge needle attached to a 1 mL syringe into a 0.25 M CaCl_2_ solution, resulting in the instant gelation of the mixture and the formation of uniform gel microfibres. To ensure the full gelation of the microfibres, they were left in the CaCl_2_ bath for several hours prior to conducting the photodegradation experiments.

### ZnO tubes and ZnOAlg characterization

The formation of ZnO tubes was confirmed using a scanning electron microscope (SEM; Quanta FEG 450). The crystallinity of the ZnO tubes was characterized using an X-ray diffractometer (XRD; Rigaku, Ultima IV) operating at a wavelength of *λ* = 1.5418 Å and 2*θ* (degree) in the range of 5–80°. A short ZnO tubes/alginate microfibre was also imaged using an environmental SEM (ESEM; Quanta FEG 450) to ensure a good distribution of ZnO tubes.

### MB photocatalytic degradation experiment

To evaluate the photocatalytic properties of cross-linked ZnOAlg microfibres, multiple 1 mL samples of ZnO and sodium alginate mixture were gelled and soaked in 0.25 M CaCl_2_ solution. A stock solution of 5 mg L^−1^ MB was freshly prepared and characterized immediately using ultraviolet, visible, near infrared (UV-Vis-NIR) spectrometer (Agilent Cary 5000). The stock solution was stored in the dark to minimize photodegradation. To prepare for the experiment, each of the 1 mL ZnOAlg microfibres samples was individually washed with deionized water to remove any residual CaCl_2_ and placed in a separate glass vial. Each vial was filled with 10 mL of the MB solution, capped, laid horizontally, and exposed to solar irradiation using a sun-solar simulator (ABET Technology Sun 2000) with a power flux calibrated to 1000 W m^−2^ (1 SUN) using Newport Oriel 91150V reference cell. Each of the prepared vails was exposed to solar irradiation for different period of time in increasing steps, starting with one vial for 30 minutes, followed by another for 60 minutes, then 90 minutes, and so on, up to a maximum of 300 minutes. All vials were kept in the dark prior to each exposure. One control vial contained MB and ZnOAlg microfibres was also kept in the dark and was not exposed to the sun simulator irradiation at all. The MB solution in each vial was characterized using the UV-Vis-NIR spectrometer immediately after the irradiation. Finally, the control vial, as well as the MB solution that was not mixed with the ZnOAlg microfibres and was kept in the dark, were also characterized to obtain the baseline results. All experiments were conducted at ambient temperature.

The photocatalytic degradation rate is determined using the following equation:
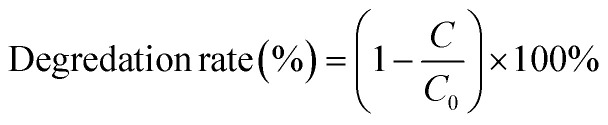
where *C*_0_ is the initial concretion of MB and *C* is the MB concentration after irradiation using sun simulator for the period of *t*.^10^

### Cyclic photocatalytic degradation experiment

To test the reusability of ZnOAlg microfibres in photocatalytic degradation, the same experiment is repeated five times using one sample of the same microfibres. Prior to each exposure to solar simulator irradiation, the microfibres were soaked in a 0.25 M CaCl_2_ solution for at least 1 hour to maintain their integrity, washed with DI water, and reused. For each cycle, the microfibres were placed in a glass vial containing 10 mL of a 5 mg L^−1^ solution of MB that had not been previously exposed to irradiation. The glass vial was then exposed to solar irradiation using the sun solar simulator, with a power flux calibrated to 1000 W m^−2^, for 5 hours. The photocatalytic degradation efficiency was determined using the same equation as in the previous experiment.

## Results and discussion

### Structural and morphological analysis

**Fig. 1:**
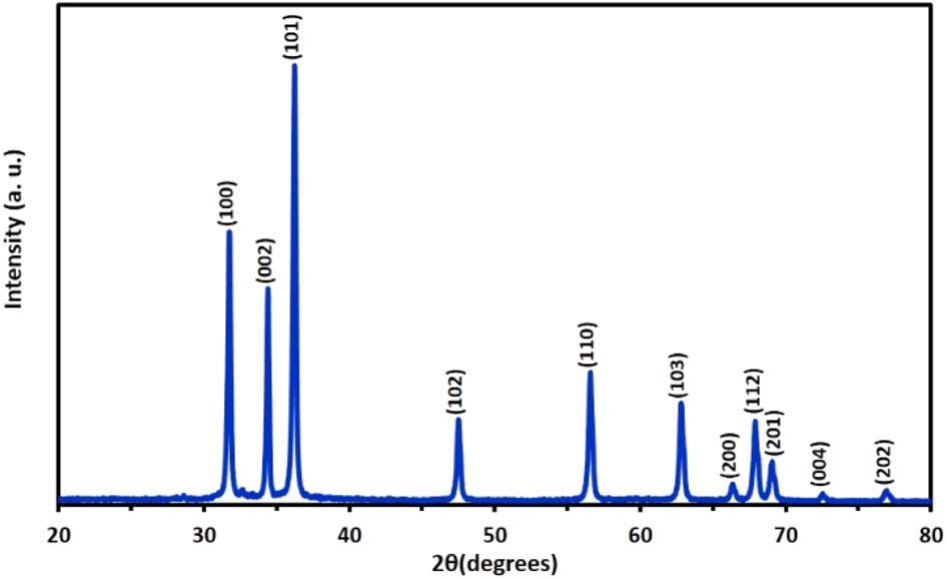
XRD pattern of the ZnO tubes with diffraction peaks confirming the presence of a hexagonal wurtzite crystal structure.

The results obtained from the XRD analyses and SEM images confirm the formation of ZnO tubes and provide insights into their structural and morphological properties. The XRD pattern presented in Fig. 1 indicates the presence of a hexagonal wurtzite crystal structure with diffraction peaks at 2*θ* values of 31.7°, 34.4°, 36.2°, 47.5°, 56.6°, and 62.8°, corresponding to the (100), (002), (101), (102), (110), and (103) Miller indices, respectively. These diffraction peaks are agree with the standard JCPDS card number 36-1451, confirming the synthesis of ZnO tubes with high quality.

Some structural properties of these strong diffraction peaks are presented in Table 1. As seen from the table, while the crystal sizes vary for each peak, the largest crystal size was calculated as 39.53 nm for 34.4° associated with the (002) plane. Strain and dislocation density values are critical parameters affecting the structural integrity of materials. While strain refers to deformation within a material owing to external forces and lattice defects, dislocation density shows the concentration of crystal defects, affecting material strength and overall stability.^48^ As presented in the table, the large size of ZnO crystals tends to reduce strain and dislocation density, resulting in lower internal stress and fewer defects. Conversely, smaller crystal sizes often correlate with higher strain and dislocation density values due to the increase in lattice distortion and higher surface area-to-volume ratio. Additionally, as the angle of the ZnO peaks increases, the *d*-spacing decreases, which is consistent with Bragg's law for X-ray diffraction.

**Table 1:** Some structural properties of ZnO tubes

2*θ* (degree)	Crystal size (nm)	D-spacing (Å)	Dislocation density (nm^−2^)	Microstrain ×10^−3^
31.7	34.38	2.82	0.8459	3.6857
34.4	39.53	2.61	0.6398	2.9668
36.2	33.56	2.48	0.8880	3.3239
47.5	28.40	1.91	1.2397	3.0301
56.6	27.04	1.62	1.3670	2.7043
62.8	26.59	1.48	1.4140	2.5003

**Fig. 2:**
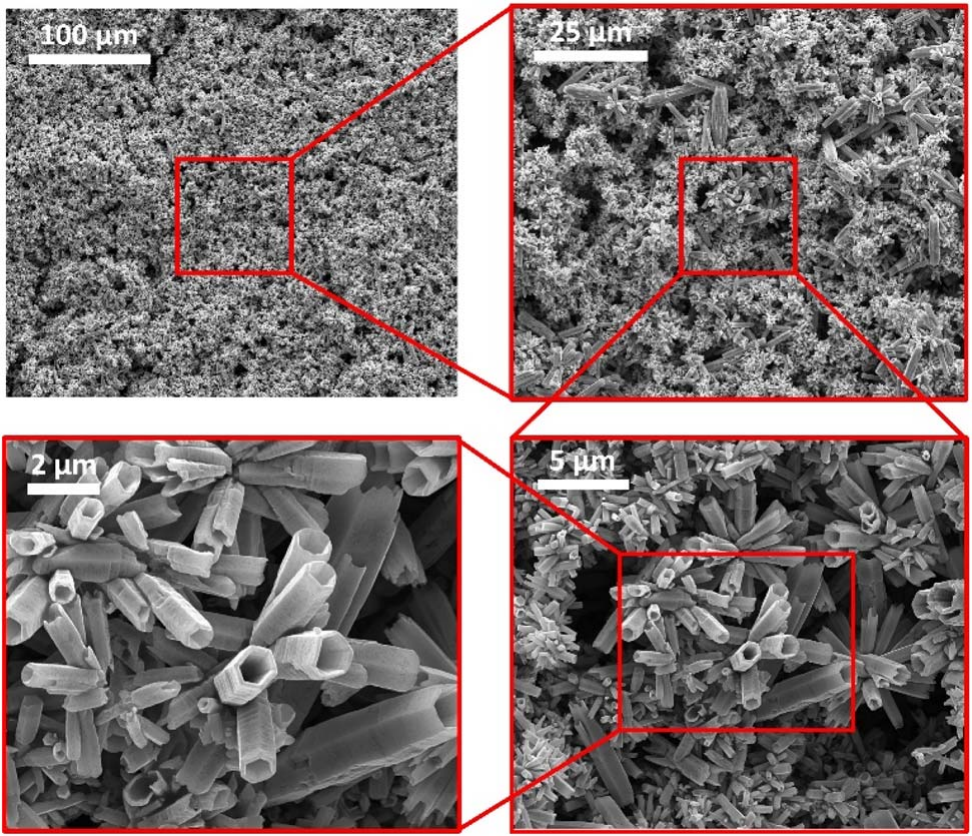
SEM images of the synthesized ZnO tubes and close-ups showing the hollow tubes structure.

The SEM images in Fig. 2 also confirm the synthesis of ZnO tubes and show that they exhibit a well-defined hexagonal tubular morphology with diameters reaching several hundreds of nanometres and a length up to tens of micrometres. Furthermore, the images show no noticeable impurities, confirming the effectiveness of the washing and filtration procedure.

**Fig. 3:**
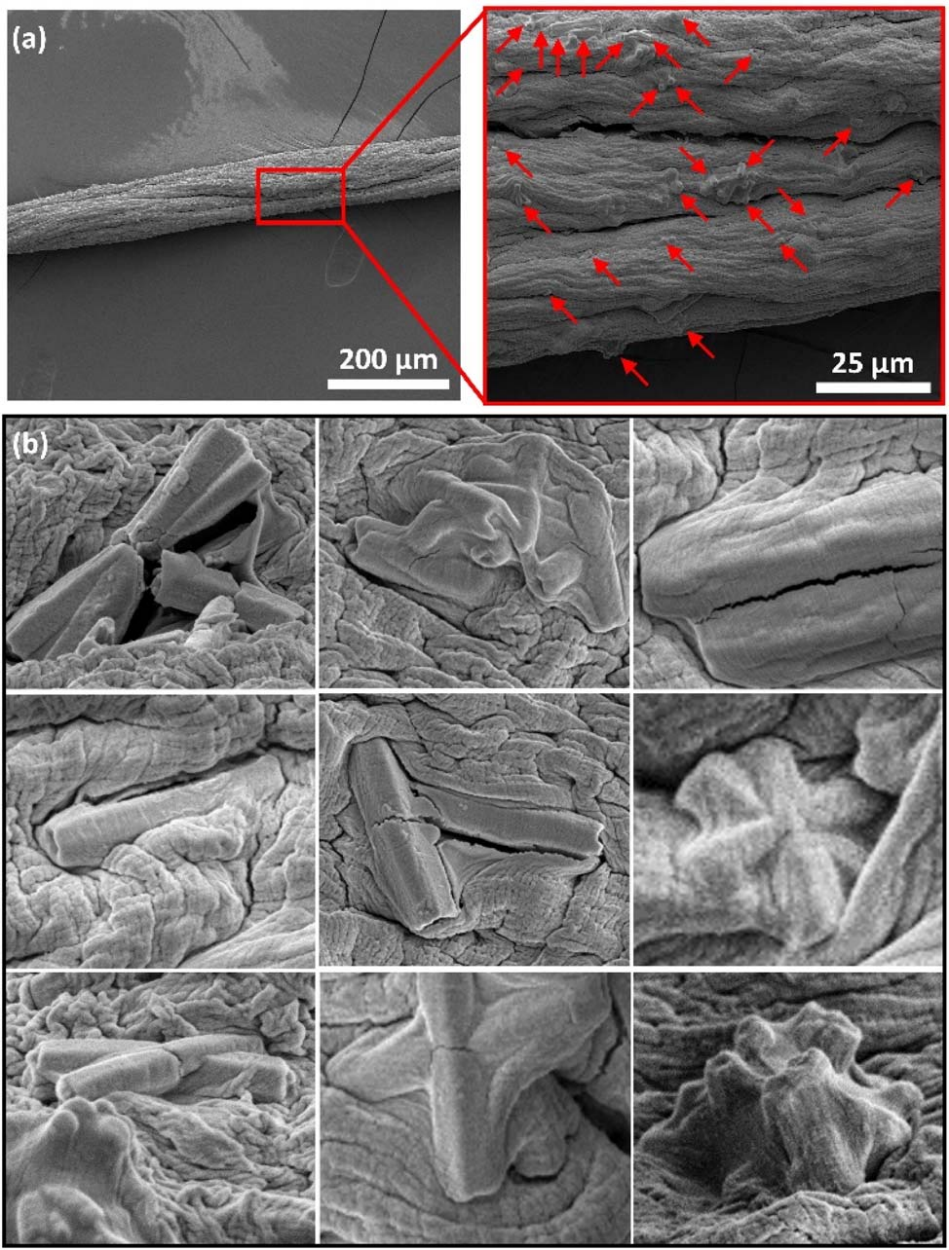
(a) ESEM image of the ZnOAlg microfibres with a close-up revealing entangled ZnO tubes highlighted by red arrows, and (b) an array of zoomed-in images depicting instances of entangled ZnO tubes.

The composite material of ZnO tubes embedded in alginate microfibres (ZnOAlg) was imaged by ESEM in Fig. 3. The ESEM images of the composite material indicate the presence of well-distributed ZnO tubes within the alginate microfibres. The alginate microfibres appeared as thin and long fibres, with an approximate diameter of around 100 μm. This diameter is consistent with the used needle gauge of 30 G, which has an inner diameter of 159 μm. The difference in the two diameters can be attributed to the shrinkage of the alginate microfibres upon gelation once extruded in the CaCl_2_ solution. The small diameter of the microfibres offers a high surface area to volume ratio, which is advantageous for the photocatalytic degradation performance of the ZnO tubes. This is because the high surface area of the microfibres can promote the efficient generation of reactive oxygen species and other photocatalytic activities. Furthermore, it allows for fast diffusion of the MB into the microfibres, facilitating the direct contact between MB and the embedded ZnO tubes.

While the ZnO tubes were observed to be distributed within the alginate microfibres, forming a network structure, some tubes were found to be tangling from the microfibres surface, which can further increase the effective surface area for photocatalytic reactions. These tubes protruding from the microfibres surface may serve as better reaction sites compared to those that are fully embedded within the microfibres, as they can more readily interact with the target pollutants in the solution. Despite the expected slight setback in photocatalytic degradation performance due to embedding the ZnO tubes within the alginate, the long and interconnected nature of the microfibres facilitates the handling and removal of the composite material after use; preventing secondary contamination and making it a suitable material for practical applications in water decontamination.

### Photocatalytic degradation results

**Fig. 4:**
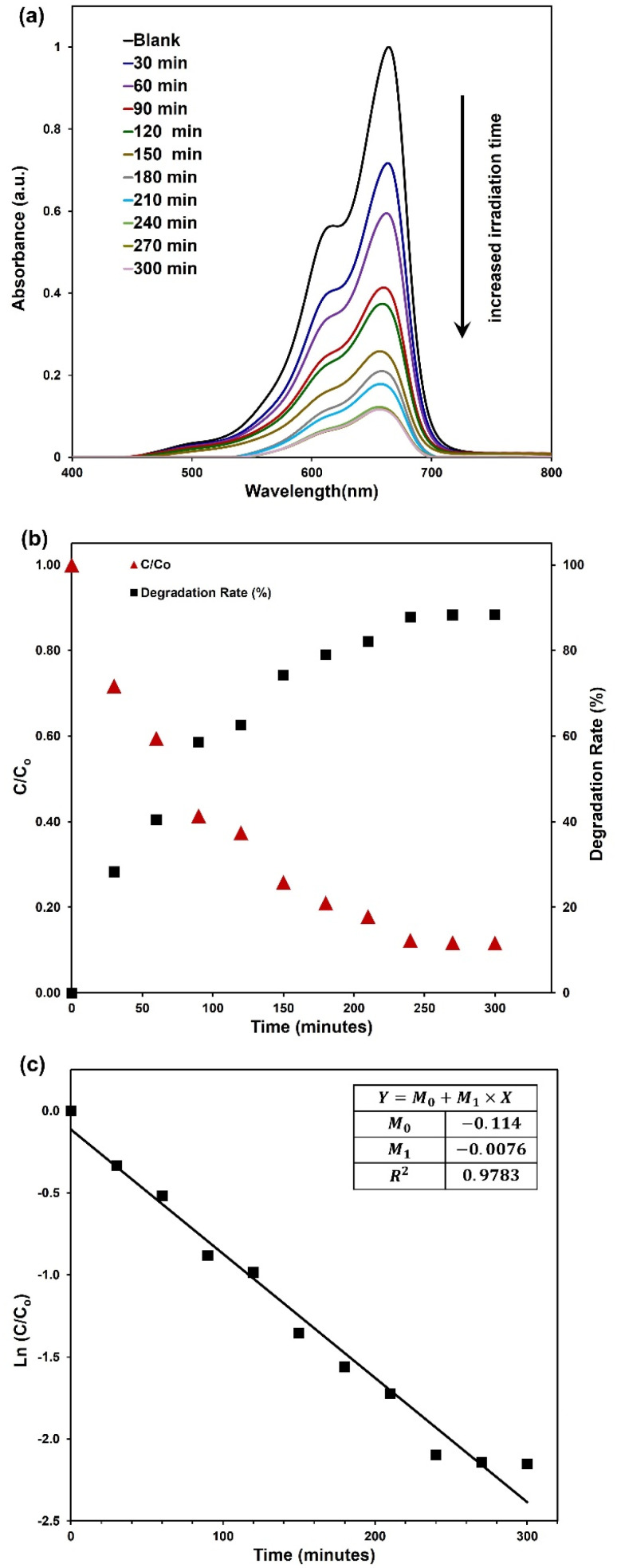
(a) plot of spectra of MB after irradiation using a sun simulator for different intervals, (b) plots of normalized MB concentration and MB degradation rate against irradiation time, and (c) plot of normalized MB concentration first-order reaction kinetics.

The photocatalytic degradation of MB existed with ZnOAlg microfibres, triggered by irradiation from the solar simulator over multiple time periods, is depicted in Fig. 4(a). Notably, in all samples, the absorption peak of MB at 663 nm demonstrates a gradual decrease in intensity with prolonged irradiation, as visually represented in the same figure. This observation confirms the progressive photocatalytic degradation of MB with increased irradiation time.

To delve deeper into the analysis of the results, we have plotted the degradation rate and MB concentration relative to the initial MB concentration (*C*/*C*_0_) for different irradiation times in Fig. 4(b). Notably, the most significant step in MB degradation, accounting for a 28% reduction, is associated with the initial 30 minutes of irradiation. It is possible that during the initial phase of irradiation, the ZnOAlg microfibres rapidly generate active species, leading to a robust photocatalytic response. Over time, there is a possibility that the ZnOAlg microfibres activity might reach equilibrium, leading to a decrease in its effectiveness in degrading MB. Furthermore, the observed decrease in efficiency could be attributed to the reduction in the concentration of MB in the solution as the process progresses. The initial high reactivity of the catalyst may result in a significant decline in MB concentration, affecting its availability for further degradation. This interaction between the catalyst's performance and the diminishing pollutant concentration could explain the fluctuations in degradation efficiency observed during extended irradiation period.

Regarding the overall efficiency of photocatalytic degradation, it is noteworthy that efficiency increases progressively with longer irradiation time, reaching approximately 88% after 300 minutes of irradiation. Notably, the curve exhibits a tendency to plateau towards the end of the observed time period, indicating a potential saturation or equilibrium in the photocatalytic degradation process.

As for the kinetics of the photocatalytic degradation, Fig. 4(c) plots ln(*C*/*C*_0_) against irradiation time, enabling the calculation of the degradation rate constant (*k*) using the kinetic equation ln(*C*/*C*_0_) = *k* × *t*. The slope of the fitted line, representing the degradation rate constant (*k*), is determined to be −0.0076 min^−1^.

It is crucial to note that various studies primarily present data on photocatalytic degradation efficiency over time and related measurements, posing challenges for direct comparisons. Notably, a significant number of these studies omit reporting the energy dose applied during their experiments, focusing instead on degradation efficiency as a function of exposure time. This oversight neglects the crucial role of the irradiated surface area. While the commonly reported metrics offer valuable insights, they do not provide a comprehensive picture of the photocatalytic activity of the material under study. To enhance the accuracy of comparisons between different photocatalytic systems, researchers should include the energy dose in the reported parameters. This additional information, combined with the typically reported metrics, facilitates a more thorough understanding of photocatalytic efficiency under specific irradiation conditions.

**Fig. 5:**
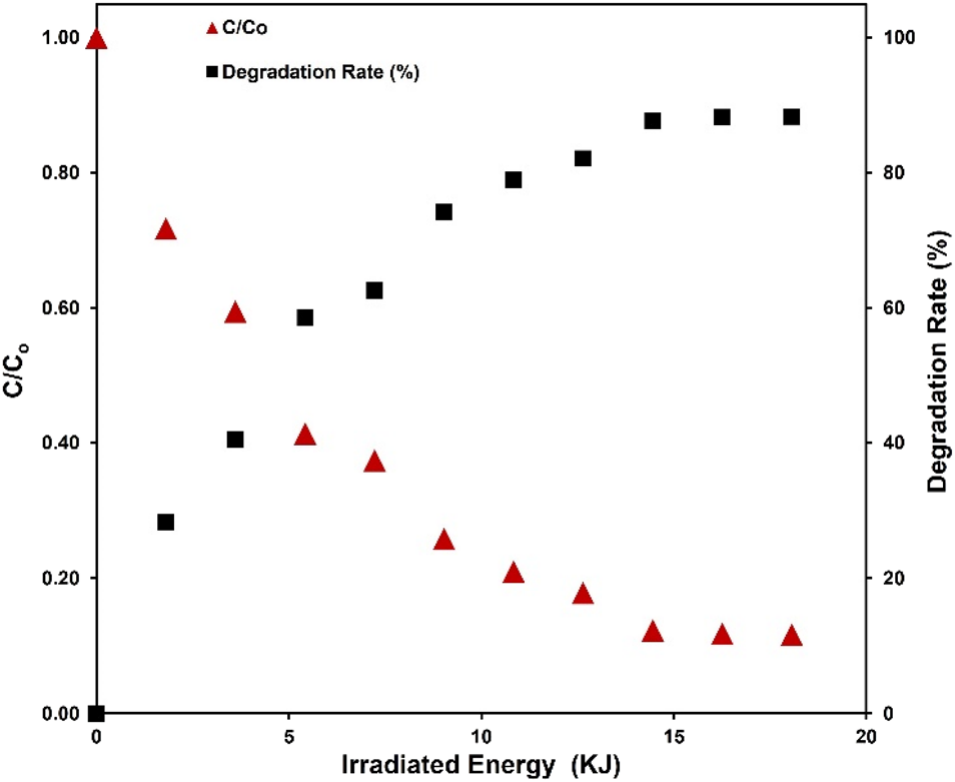
Plots of normalized MB concentration and MB degradation rate against irradiated energy.

To address this concern, we propose an innovative approach by introducing the plotting of the normalized pollutant concentration against irradiated energy. This representation effectively consolidates various influential parameters into a single plot, in which the *x*-axis representing the irradiated energy (*E*_irr_) encompasses factors such as irradiation power flux, irradiated surface area, and irradiation time. This approach is demonstrated in Fig. 5, which also shows that the 88% MB degradation is achieved after irradiation the sample with approximately 18 kJ. Additionally, we introduce a new metric, termed ‘Specific Energy Efficiency’ (SEE), expressing the ratio of the degraded mass of the pollutant to the mass of the photocatalytic active material for each unit of irradiated energy. The SEE metric is defined by the equation:
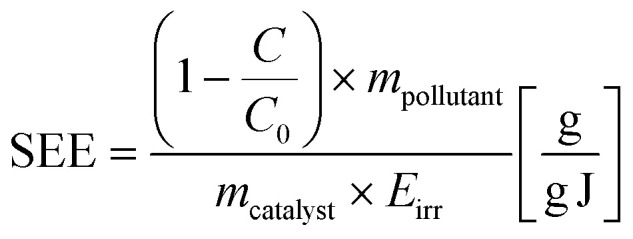
Here, *m*_pollutant_ represents the initial mass of the pollutant in the sample, making the numerator the total degraded pollutant mass after irradiation. *m*_catalyst_ denotes the mass of the photocatalytic active material, and *E*_irr_ stands for the irradiated energy. Notably, this equation along with plotting against irradiated energy rather than time, as in Fig. 5, render the results independent of the experimental setup, enabling comparability across studies. Therefore, we recommend reporting both the SEE and other plots that illustrate the degradation ratio *vs.* irradiated energy. In our experiments, we found that the maximum SEE is 1.044 μg g^−1^ J^−1^, and the average SEE for the entire experiment is 326 ng g^−1^ J^−1^.

### Cyclic photocatalytic degradation results

The results of the photocatalytic degradation cyclic experiments initially demonstrated consistent and stable performance, achieving substantial MB degradation ranging from 81% to 89% under the prescribed conditions. However, a significant decline in performance was observed in the fifth cycle, with MB degradation dropping to 72%. Notably, by the conclusion of the same cycle, the microfibres exhibited a loss of structural integrity, breaking down into shorter microfibre pieces.

The most likely explanation for this phenomenon involve the intricate interplay of chemical changes induced by repeated cycles and thermal changes originating from irradiation by sun simulator radiation. Repeated photocatalytic degradation processes may have caused chemical changes in the microfibres, contributing to its structural and efficiency degradations. Simultaneously, the impact of thermal changes may have worsened these effects, further degrading the mechanical integrity of the microfibres. While these factors may have individually contributed to the observed outcomes, their combined impact likely played a role in both the diminished photocatalytic performance and the breakdown of the microfibres.

Despite this decrease in photodegradation efficiency in the fifth cycle, the photocatalytic activity remains significant and does not undermine the overall potential of the composite. The alginate can be dissolved, and the ZnO tubes can be recovered and reused to form new microfibres. This concept has been previously demonstrated in the literature. For example, Zhao *et al.* embedded TiO_2_ nanoparticles in alginate and showed that TiO_2_ nanoparticles could be recovered after use by dissolving the alginate in a sodium citrate solution.^11^ Furthermore, the stability observed over the first 4 cycles is promising. This trade-off is worthwhile given that the composite prevents secondary contamination of water, a significant practical advantage for water decontamination applications. The ability to recycle and reuse the ZnO nanotubes further enhances the practicality and sustainability of this approach.

## Conclusions

This study showed the effectiveness of ZnOAlg microfibres in the photocatalytic degradation of MB. A substrate-free hydrothermal process was employed in the synthesis of the ZnO tubes. The incorporation of these tubes into calcium alginate microfibres, chosen for the microfibres expansive surface area. This was not only advantageous for the photocatalytic degradation performance but also facilitated the microfibres collection after the decontamination process, preventing secondary contamination of the water with ZnO tubes. Structural and morphological analyses using XRD and SEM, was used to characterize the properties of the ZnO tubes and the resulting composite material. Validation of the composite's efficacy was evident through the degradation of MB, achieving an impressive 88% degradation under irradiation of approximately 18 kJ in 5 hours. Cyclic tests consistently demonstrated significant degradation levels in the initial four cycles (81–89%), followed by a subsequent decreased performance and microfibres breakdown into shorter fragments. This dynamic behaviour highlights the nuanced nature of the photocatalytic process and emphasizes the need to consider long-term performance.

This study also introduced a novel reporting methodology, plotting normalized concentration against irradiated energy, and introduced the SEE metric. These innovations addressed existing limitations in reporting methods, offering valuable enhancements for result comparability across studies. They contribute to the establishment of a standardized framework for future research in the field.

## Conflicts of interest

There are no conflicts to declare.
